# The History and Capabilities of Herbal Simples

**Published:** 1893-10-28

**Authors:** W. T. Fernie


### THE HISTORY AND CAPABILITIES OF HERBAL SIMPLES.

By W. T. Fernie, M.D.

(*Continued from page* 420., *Vol. XIV.*)

### GOOD KING HENRY.

Growing very commonly in waste places near our English villages, and having undoubted curative virtues, one of the goosefoot tribe, the Good King Henry, should certainly find mention among herbal simples. It has really nothing to do with our Harry the Eighth, of pious memory, and his sore legs, though many persons suppose its name to be derived from such an association. This Chenopod—so named from "keen," a goose, and "pous," a foot—is a succulent plant of mealy aspect, and about a foot high, with dark green, triangular, thickish leaves, which have a salt, mucilaginous taste, and are cooked in some places instead of spinach. Our beetroot belongs to the same order of plants. This particular goosefoot, the Good King Henry, is called—"bonus," good—to distinguish it from another goosefoot, the "malus," bad, or poisonous. The title Henricus comes from the German name of the plant—"Heinrich," an elf or goblin, as indicating a supposed possession of magical powers. In some districts the plant is known as blite, or tasteless, and, as says Evelyn in his "Book of Salads," "'Tis insipid enough!"

This, as well as the other goosefoots, affords much soda. It has been also called fat hen, because used as food for poultry both here and in Germany. An allied American goosefoot produces active and popular worm seeds for expelling round worms. Another variety, the Stinking goosefoot, or dog's Orach (Arach, or golden herb), is found freely in waste places, and on roadsides in England, especially near the sea. Its very disgusting odour is like that of putrid fish, and its ashes yield a large quantity of potash. This plant has become famous for its efficacy in hysterical maladies. The leaves are made with sugar into a conserve, and Dr. Fuller prepared from them a noted electuary, adding some oil of amber, and giving with much benefit a piece as big as a chestnut for any hysterical spasms. But verily this simple has a "most ancient fish-like smell," which is *odoris virosi intolerabilis*, and which clings to the hands long after touching the herb. Chemically it contains much "trimethalymine"—a capital Abracadabra to conjure with.

The Good King Henry, or Allgood, bears small flowers of sepals only; it is grown in Lincolnshire as a pot herb, which cottagers call Mercury. The young shoots are gently laxative, the leaves are boiled in broth, and the roots are given to sheep afflicted with cough. Poultices made from the plant are applied to cleanse and heal old ulcers, which—as Gerarde tells us—they do scour and mundify. Small wonder is it that readers of history have connected Henry VIII. with the probable use of so salutary a remedy. His bodily infirmities towards the close of his life were sorely desperate. We are told he became in mind and person a most loathsome object: the ulcers in his leg had grown revoltingly offensive; his weight and helplessness were such that he could not pass through any ordinary door nor be removed from one part of the house to another except by the aid of machinery, and with the help of numerous attendants. The constant irritation of his festering legs made his irritable temper still more terrible."

The title—Orach—given to the Stinking Goosefoot, and to others of the same tribe, is a corruption of *aurum*, gold, because their seeds were supposed to cure the disease popularly known as the yellow jaundice:—

With vinegar, honey, and salt, the Orach
Made hot and applied, cures a gouty attack;
While its seeds, for the jaundice, if given in wine,
As Galen has said, are a remedy fine.

The goosefoot plants, including Good King Henry, get their Greek name *Chenopod* (also meaning goosefoot), from the likeness which their leaves bear to the webbed extremities of the waddling bird.

Our raw military recruits, when beginning to drill, make an irksome acquaintance with this halting gait known as the goose-step.

